# Spatial intra-tumour heterogeneity in acquired resistance to targeted therapy complicates the use of PDX models for co-clinical cancer studies

**DOI:** 10.15252/emmm.201505431

**Published:** 2015-07-14

**Authors:** Claudia Wellbrock

**Affiliations:** Manchester Cancer Research Centre, Wellcome Trust Centre for Cell-Matrix Research, University of ManchesterManchester, UK

## Abstract

Targeted therapy in the treatment of cancer has produced great clinical successes. However, with these came the challenge of acquired resistance. Melanoma, a cancer that carries one of the highest mutational burdens, displays great complexity in mutational acquired resistance with a notable degree of inter-tumoural heterogeneity. In this issue of *EMBO Molecular Medicine*, Kemper *et al* (2015) describe the identification of multiple, partly novel resistance mechanisms present in one patient and within a single metastasis, where one mutation could be traced back to a pre-treatment lesion. Importantly, the observed intra-tumoural “spatial” heterogeneity can impact on the interpretability of patient-derived xenografts, and this might have implications particularly for co-clinical treatment studies.

See also: **K Kemper *et al*** (September 2015)

Melanoma is a skin cancer originating from melanocytes, pigment cells whose proliferation and differentiation programmes are governed by the ERK/MAP-kinase pathway (Wellbrock *et al*, 2002). Targeting the MAP-kinase pathway component BRAF, which carries activating mutations in ∼50% of melanomas (Davies *et al*, 2002), has revolutionized the treatment of this cancer. However, acquired resistance is frequent, and the subsequent dissection of underlying mechanisms led to the discovery of various novel interactions and regulatory connections within the MAP-kinase signalling network in melanoma cells. Amongst these are non-genetic events such as deregulation of receptor tyrosine kinases or the MEK-kinase MAP3K8, disrupted feedback regulation, stromal secretion of growth factors and alternative splicing of *BRAF* RNA transcripts. In addition, a variety of genetic causes, which include *NRAS* and *MEK* mutations, *BRAF* amplification and loss of *NF1*, have been identified (Shi *et al*, 2014; Van Allen *et al*, 2014). Strategic whole-exome and cDNA sequencing has revealed that up to 70% of tumours progress with mutations that lead to ERK reactivation in the presence of drug (Shi *et al*, 2014; Van Allen *et al*, 2014), hinting at an enormous advantage of active MAP-kinase signalling for melanoma cell growth. In addition, significant intra-tumoural mutational heterogeneity has been observed in melanoma lesions (Shi *et al*, 2014; Van Allen *et al*, 2014), clearly indicating that melanoma is a malignancy with profound mutational burden.

In this issue of *EMBO Molecular Medicine*, Kemper *et al* (2015) unveil yet more complexity by identifying additional novel resistance mechanisms, which display “spatial” intra-tumour heterogeneity. The Peeper group analysed multiple drug-resistant metastases from one patient and found them to all display increased phosphorylation of ERK, thus revealing consistency in pathway reactivation. However, the underlying resistance mechanisms were strikingly different. The authors confirm inter-tumour heterogeneity amongst distinct metastases of a single patient and identify three independent *BRAF* amplification events, a novel aberrant form of BRAF^V600E^ that contains an unknown N-terminus, and a novel MEK1 mutation, which was detected in two out of five metastases.

Of particular note is a MEK1 mutation, namely a three-base pair in-frame insertion in exon 2, which probably causes a conformational change in the protein and renders MEK1^T55delinsRT^ constitutively active. This mutation was detected in two of the five analysed metastases. What is remarkable is that the mutation produces a MEK protein that *per se* reduces cell-autonomous growth, but allows cells to thrive in the presence of drug and as such confers resistance to BRAF and ERK inhibitors. This suggests that MEK1^T55delinsRT^ mutant cells might have been enriched in the respective metastases on treatment. Nevertheless, the growth of cells expressing the MEK1^T55delinsRT^ mutant can still be blocked by MEK inhibition but strikingly, although several MEK inhibitors (including PD-032590, U0126 and selumetinib) were tested, only trametinib was efficient in killing the cells. Because all tested inhibitors act in a non-ATP-competitive manner, this suggests that the efficacy of trametinib is due to inhibitor potency, not mode of action. This is important, because it suggests that if the potency of MAPK-pathway inhibitors is increased (possibly through combination therapies), there could be room for improvement in the treatment of patients with advanced melanoma even if they relapse with pathway reactivation.

Interestingly, in one metastasis the *MEK1*^*T55delinsRT*^ mutation was found to coexist with a *BRAF* amplification (Fig1). This particular lesion still responded to treatment, and the authors suggest that selection for a dominant clone providing acquired resistance might have still been ongoing. However, selection for one clone might not be necessary to reach acquired resistance. For instance, concurrent *NRAS* and *MEK1* mutations or even two different *NRAS* mutations have previously been identified to be present within the same tumour on progression (Van Allen *et al*, 2014). Since kinase inhibitors are unlikely to produce mutations *per se*, this raises the possibility that individual genetically different sub-populations pre-exist and, rather than competing, they co-operate in the presence of drug. Indeed, the *MEK1*^*T55delinsRT*^ mutation was not only present in two metastases, but was also found to pre-exist before treatment.

**Figure 1:**
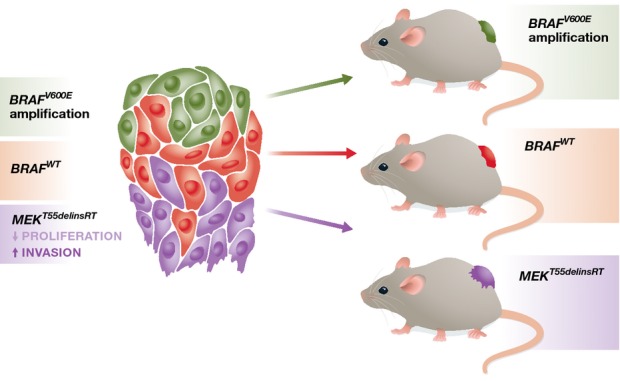
Intra-tumour mutational heterogeneity challenges PDX studies Intra-tumour mutational heterogeneity occurs in melanoma, where *MEK1*^*T55delins*^^*RT*^*-*positive cells (purple) were found to coexist with cells harbouring a *BRAF* amplification (green) within one tumour of a patient on vemurafenib treatment. The *MEK1*^*T55delins*^^*RT*^ mutation was also identified in a tumour before treatment, but because in the absence of drug MEK1^T55delins^^RT^-expressing cells feature reduced cell growth, they might have to co-operate with other cell populations within the tumour to gain fitness. On the other hand, MEK1^T55delins^^RT^-expressing cells are more invasive and other cell populations within the heterogeneous tumour could take advantage of this property and co-invade. Taking small fragments from such a heterogeneous tumour to grow a PDX can result in different outcomes, because spatial heterogeneity will lead to the prevalence of different mutations in separate PDX models.

Because MEK1^T55delinsRT^ suppresses cell-autonomous growth, the existence in a population of cells before treatment is unexpected. On the other hand, it may be that in the heterogeneous tumour environment, *MEK1*^*T55delinsRT*^-positive cells could interact with and receive growth support from other genetically differing cells; in turn, *MEK1*^*T55delinsRT*^ cells might provide similar support to other sub-clones. In this context, it is striking that the *MEK1*^*T55delinsRT*^ mutation rendered melanoma cells more invasive (Fig1). While it is not known whether this would provide an advantage for cells with a *BRAF* amplification, such co-operative behaviour has been observed before, whereby invasive, less proliferative cancer cell sub-populations co-operate with non-invasive, highly proliferative sub-populations to enhance tumour fitness (Chapman *et al*, 2014).

An important finding made by Kemper *et al* (2015) is that intra-tumour heterogeneity impacts on the interpretation of patient-derived xenograft (PDX) studies. This became obvious when the group analysed distinct fragments of the original lesion and obtained different results regarding the mutational alterations that were detectable in these biopsies (Fig1). The great advantage of PDX models lies in the fact that they consider the full tissue context, as the local tumour microenvironment including cellular heterogeneity is maintained (Siolas & Hannon, 2013). PDXs are usually derived as small tumour fragments from larger heterogeneous lesions, but this is where spatial heterogeneity can create a problem. The study shows that PDXs can cover inter-tumour heterogeneity, thus confirming that *per se* PDXs can be used as surrogates to study tumour biology and therapy response. But the spatial heterogeneity derived from intra-tumour heterogeneity makes it more difficult to capture the full extent of genetic heterogeneity seen in an original tumour.

In conclusion, the findings presented by Kemper *et al* (2015) do suggest that we cannot safely assume that every PDX fully reflects the genetic make-up of the corresponding tumour in a patient. This might cause a problem, particularly with regard to co-clinical treatment studies, where conclusions are often drawn from one single PDX per patient. While intra-tumour heterogeneity with regard to mutations conferring resistance to BRAF inhibitors has been reported before (Shi *et al*, 2014; Van Allen *et al*, 2014), the study by the Peeper group is the first to highlight the possible complications for PDX studies. It should however be noted that the study analyses only one patient and encounters the problem of spatial heterogeneity for the PDX system in only one metastasis. Furthermore, for this particular sample, the detection of the two individual genetic alterations was low. Thus, the mutations might have been present throughout the whole tumour and only increased sensitivity in the analysis would have been able to identify them uniformly. Thus, further studies will have to confirm whether the frequency of spatial intra-tumour heterogeneity is high enough to have a significant impact on the conclusions drawn from PDX studies. Nevertheless, the profound mutational heterogeneity that is found in acquired resistant tumours challenges the current idea that DNA sequence information (from biopsies, or circulating DNA/tumour cells) will be conclusive enough to inform decisions with regard to salvage therapies in progressed patients with acquired resistance.
